# Evaluation of cytotoxicity and corrosion resistance of orthodontic mini-implants

**DOI:** 10.1590/2177-6709.21.5.039-046.oar

**Published:** 2016

**Authors:** Celha Borges Costa Alves, Márcio Nunes Segurado, Miriam Cristina Leandro Dorta, Fátima Ribeiro Dias, Maurício Guilherme Lenza, Marcos Augusto Lenza

**Affiliations:** 1 PhD student, Universidade Federal de Goiás, School of Dentistry, Department of Clinical Dentistry, Graduate Program in Dentistry, Goiânia, Goiás, Brazil.; 2 Professor, Universidade Paulista (UNIP), School of Dentistry, Department of Orthodontics, Goiânia, Goiás, Brazil.; 3 Associate Professor, Universidade Federal de Goiás (UFG), Institute of Tropical Pathology and Public Health, Immunology Section, Department of Microbiology, Immunology, Parasitology and Patology, Goiânia. Goiás, Brazil.; 4 Full Professor and Coordinator, Universidade Federal de Goiás (UFG), School of Dentistry, Specialization Course in Orthodontics, Goiânia, Goiás, Brazil).

**Keywords:** Mini-implants, Corrosion, Cytotoxicity

## Abstract

**Objective:**

To evaluate and compare *in vitro* cytotoxicity and corrosion resistance of mini-implants from three different commercial brands used for orthodontic anchorage.

**Methods:**

Six mini-implants (Conexão(tm), Neodent(tm) and SIN(tm)) were separately immersed in artificial saliva (pH 6.76) for 30 and 60 days. The cytotoxicity of the corrosion extracts was assessed in L929 cell cultures using the violet crystal and MTT assays, as well as cell morphology under light microscopy. Metal surface characteristics before and after immersion in artificial saliva were assessed by means of scanning electron microscopy (SEM). The samples underwent atomic absorption spectrophotometry to determine the concentrations of aluminum and vanadium ions, constituent elements of the alloy that present potential toxicity. For statistical analysis, one-way ANOVA/Bonferroni tests were used for comparisons among groups with *p* < 0.05 considered significant. Statistical analysis was carried out with Graph Pad PRISM software Version 4.0.

**Results:**

No changes in cell viability or morphology were observed. Mini-implants SEM images revealed smooth surfaces with no obvious traces of corrosion. The extracts assessed by means of atomic absorption spectrophotometry presented concentrations of aluminum and vanadium ions below 1.0 µg/mL and 0.5 µg/mL, respectively.

**Conclusion:**

Orthodontic mini-implants manufactured by Conexão(tm), Neodent(tm) and SIN(tm) present high corrosion resistance and are not cytotoxic.

## RESUMO

**Objetivo:**

avaliar, *in vitro*, e comparar a citotoxicidade e a resistência à corrosão de mini-implantes de três marcas comerciais diferentes, utilizados para ancoragem ortodôntica.

**Métodos:**

seis mini-implantes fabricados pelas empresas Conexão^(r)^, Neodent^(r)^ e SIN^(r)^ foram imersos, separadamente, em saliva artificial (pH = 6,76), por 30 e 60 dias, de forma a obter os extratos da corrosão. A citotoxicidade dos extratos foi avaliada em cultura de células L929, empregando-se a análise de ensaios do cristal violeta e MTT, bem como da morfologia celular sob microscopia óptica. As características da superfície do metal antes e após a imersão em saliva artificial foram avaliadas usando microscopia eletrônica de varredura. Os extratos foram submetidos a espectrofotometria de absorção atômica, para determinar as concentrações dos íons alumínio e vanádio, elementos constituintes da liga e que apresentam toxicidade em potencial. Para análise estatística, os testes *one-way* ANOVA/Bonferroni foram usados para comparação entre os grupos, com *p* < 0,05 sendo considerado significativo. A análise estatística foi realizada com o programa Graph Pad PRISM v. 4.0.

**Resultados:**

não foi observada alteração na viabilidade ou morfologia celular após a exposição dos mini-implantes aos extratos. A análise dos mini-implantes por microscopia eletrônica de varredura revelou superfícies lisas e sem traços evidentes de corrosão. Os extratos analisados usando espectrofotometria de absorção atômica apresentaram concentrações de íons alumínio e vanádio inferiores a 1,0 µg/ml e 0,5 µg/ml, respectivamente.

**Conclusão:**

os mini-implantes fabricados pelas empresas Conexão^(r)^, Neodent^(r)^ e SIN^(r)^ apresentam alta resistência à corrosão e não são citotóxicos.

## INTRODUCTION

Understanding each patient's anchorage requirements is extremely important and ensures high-quality orthodontic treatment. If anchorage is lost, it will undoubtedly result in compromised results. Relying on patient's compliance to obtain the desirable force system will also increase the risk of not achieving the desirable finishing results. Today, mini-implants provide the much-desired absolute anchorage and, more importantly, the use of these devices does not rely on patient's compliance. They are used primarily as direct or indirect anchorage - a biomechanical setup in which force is directly or indirectly applied from the mini-implant to a tooth or a group of teeth that needs to be orthodontically moved. Therefore, in the last few years, mini-implants have been extensively used for anchorage, thus simplifying orthodontic mechanics and minimizing side effects during orthodontic treatment.^1^ ^-^ ^5^

The ongoing and continuous use of metal material in Orthodontics has led to a large number of laboratory and clinical studies on the detrimental effects of corrosion products to one's general health. The oral cavity is not only extremely aggressive, but also a potential corrosive environment. Corrosion resistance of orthodontic alloys depends on the oral environment which is influenced by several variables, such as the amount and quality of saliva, pH of food and beverages, among others.^6^ The release of metal ions from orthodontic devices is a genuine concern. 

Although all types of metallic material are subject to corrosion, titanium is widely used in orthopedic components because of its attractive characteristics, such as high corrosion resistance and excellent biocompatibility. Additionally, it presents excellent mechanical properties and provides resistance to stress and strain. It is, therefore, considered an ideal material.^7^ ^-^ ^11^ However, pure titanium has less fatigue strength than titanium alloys. Orthodontic mini-implants should withstand high orthodontic loads for tooth movement. In order to overcome potential fractures of commercially pure titanium during mini-implant placement and removal, aluminum and vanadium have been added to the alloy for greater strength and fatigue resistance.^12^ ^,^ ^13^

Titanium alloy (Ti_6_Al_4_V) is now most often used in Dentistry to overcome this disadvantage. However, this alloy may undergo corrosion in the oral environment due to its low corrosion resistance. Titanium, aluminum and vanadium ions can be released to local and remote tissues and have been associated with side effects in the human body, particularly aluminum and vanadium.^14^ ^-^ ^18^

Although *in vitro* studies do not reproduce the complex oral environment, standard assays are useful to evaluate the cytotoxicity and biocompatibility of temporary anchorage devices, such as mini-implants. ISO 10271:2011 provides test methods to determine the corrosion behavior of metallic material used in the oral cavity.^19^

The aim of this *in vitro* study was to compare the cytotoxicity and corrosion resistance of mini-implants from three different commercial brands used for orthodontic anchorage.

## MATERIAL AND METHODS

### Samples 

This study investigated metal mini-implants used for orthodontic anchorage fabricated by three commercial manufacturers: Conexão^TM^, São Paulo, Brazil; Neodent^TM^, Curitiba, Brazil and SIN^TM^, São Paulo, Brazil - respectively with mini-implants head diameter and total length of 1.5 x 12 mm, 1.6 x 11 mm and 1.6 x 12 mm (Table 1). Although the exact mini-implant chemical composition was not provided by the manufacturers, they followed the standard specification for Titanium-6Aluminum-4Vanadium ELI (extra low interstitial) alloy for surgical implant applications (ASTM F136-08e1 - UNSR 56401).

**Table 1:** Commercial mini-implants evaluated in this study.

Mini-implants	Diameter	Length	Lot number	Manufacturer
Conexão(tm)	1.5 mm	12 mm	4050804208	Conexão Sistemas de Prótese, Arujá/SP, Brazil
Neodent(tm)	1.6 mm	11 mm	20768	JJ GC Indústria e Comércio de Materiais Dentários S.A. Neodent, Curitiba/PR, Brazil
SIN(tm)	1.6 mm	12 mm	C7145	SIN - Sistema de Implante Nacional S.A., São Paulo/SP, Brazil

Six samples of each orthodontic mini-implant manufacturer were individually weighed with the aid of an analytical balance (model 410 - Kern & Sohn GmbH, Balingen, Germany) and autoclaved at 120 ^o^C for 30 minutes. Subsequently, each sample was transferred to individual sterile BD Vacutainer^TM^ glass tubes (Becton Dickinson Indústrias Cirúrgicas Ltda, Juiz de Fora, MG, Brazil) and immersed in artificial saliva for 30 and 60 periods. The number of samples and methods used are in accordance to corrosion test methods for metal material specified in ISO 10271.^19^ The procedures were carried out in a laminar flow hood, with ultraviolet radiation used to obtain an aseptic field.

 The artificial saliva chemical composition used in this study was a modification of Meyer's solution^20^ ^,^ ^21^ which has been shown to present corrosion activity and chloride concentration similar to natural saliva. It was composed of 0.40 mg/L of NaCl, 0.40 mg/L of KCl, 0.80 mg/L of CaCl_2_.H_2_O, 1.0 mg/L of CO(NH_2_)^2^ in distilled water with a pH adjusted and controlled with a 10-N NaOH solution. The performance of any material placed into the oral environment should be assessed with artificial saliva of a known composition, since natural saliva varies widely.^22^

The amount of saliva was calculated by the ratio of 1 mL of artificial saliva for 0.2 g of mini-implant weight, according to ISO 10271^19^ and ISO 10993-15.^20^ Mini-implants were maintained in immersion and stored at 37 ^o^C under stationary conditions. Tubes containing only artificial saliva, without the mini-implant extract, were stored under the same conditions as negative control. 

After the immersion periods, mini-implants were removed from the tubes, washed in deionized water, dried and stored in new sterile airtight plastic tubes, and saliva with the mini-implant corrosive product extracts was stored in 1.5-mL tubes at 4 ^o^C for further analysis. The methods used in this study have already been described.^24^

### L929 cell culture

Murine fibroblast L929 cells were cultured in 75-cm^2^ culture flasks (Corning Costar Corporation, Cambridge, MA, USA) containing RPMI 1640 culture medium (Sigma-Aldrich Co. LLC, St. Louis, MO, USA), buffered with 10-mM HEPES and supplemented with 10% fetal bovine serum (FBS) (Gibco/BRL Division, Grand Island, NY, USA), 2-mM L-glutamine, 11-mM sodium bicarbonate, 100-U/mL penicillin, and 100-g/mL streptomycin (Sigma-Aldrich Co. LLC, St. Louis, MO, USA), herein named complete medium. After L929 cell monolayer formation, the culture medium was removed and the cells washed with 1 mL of incomplete medium (RPMI 1640 without FBS). The cells were detached from the culture flasks with 0.025% trypsin (Sigma-Aldrich Co. LLC, St. Louis, MO, USA).

After trypsinization, cultured cells were resuspended in 5 mL of culture medium, transferred to 50-mL plastic tubes (Corning Costar Corporation, Cambridge, MA, USA) and centrifuged at 2,000 rpm for 10 minutes at 15 ^o^C. For culture maintenance, cells were cultivated again in complete medium (? 1 × 10^5^ cells/mL). For cytotoxicity assays, cells were resuspended in 1 mL of complete medium. Viable cells were counted by trypan blue dye exclusion test (in 0.1% phosphate buffered saline) using a hemacytometer adjusted to a concentration of 3.5 × 10^5^ cells/mL by adding 0.9% NaCl.

Artificial saliva was used as negative control and as a medium to obtain mini-implant extracts, since it is not cytotoxic to cell-culture. Tumor necrosis factor (TNF, Sigma-Aldrich Co. LLC, St. Louis, MO, USA), a cytokine capable of destroying L929 cells after approximately 20 hours of culture, was used as positive control. 

### Cytotoxicity assays

Aliquots of 100 µL of L929 cell suspension were pipetted into 96-well flat bottom plates (Corning Costar Corporation, Cambridge, MA, USA). External wells were half filled and the plates incubated for 48 hours at 37 ^o^C in a humidified atmosphere with 5% CO_2_ to obtain a cell monolayer. After this period, monolayer growth was confirmed by inverted light microscope and 20-µL aliquots (20%) of mini-implant extracts or 20-µL (20%) of artificial saliva (used as negative control) were added to the correspondent well. Mini-implant extract solution was tested in triplicate on every plate and incubated for 48 hours at 37 ^o^C in a humidified atmosphere with 5% CO_2_.

**Figure 1:**
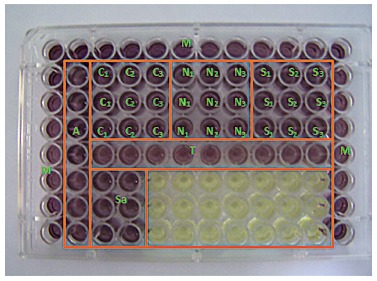
Configuration of the 96-well flat bottom plates in which the cytotoxicity assay was carried out. M = complete medium without cells. A = complete medium without extract solutions. Sa = artificial saliva (negative control). T = TNF (positive control). C1, C2, and C3 = mini-implants Conexão, tested in triplicate. N1, N2, and N3 = mini-implants Neodent, tested in triplicate. S1, S2, and S3 = mini-implants SIN, tested in triplicate. The yellow wells remained empty.

Aliquots of 100 µL of TNF solution were placed in each well of a flat bottom plate containing 100 µL of L929 cells followed by serial dilutions at a 2/1024 to 1/1024 ratio applied to the neighboring wells of the same row (Fig 1). After dilution, 10-µL aliquots of actinomycin D (20 mg/mL, Sigma-Aldrich Co. LLC, St. Louis, MO, USA) were added to each well to increase cell sensitivity to TNF (Fig 1).

### Determination of cell viability by means of the crystal violet colorimetric assay

After a 48-hour incubation period, 10-µL aliquots of 0.5% crystal violet in 30% acetic acid were added to each well to fix the living cells to the bottom of the plate. After 10 minutes, the plates were washed to have dead cells removed and, after complete drying in a bacteriological incubator at 37 ^o^C, 100 mL of absolute methanol (Synth, Diadema, SP, Brazil) was added to dissolve the stained cells. The resulting stained solution, corresponding to the total number of viable cells retained on the plates, was placed in a microplate spectrophotometer (Multiskan Original, Model 352, Thermo Labsystems, China, filter 620 nm) and optical density (OD) was read. Culture medium without cells was the blank. Control wells absorbance (cells cultured in complete medium) was considered as 100% cell viability. Results were expressed as OD.

### Determination of cellular metabolism by means of the MTT colorimetric assay

For the MTT assay, L929 cells were grown and, after 48 hours of incubation, 10-µL aliquots of MTT solution (5 mg/mL phosphate-buffered solution, PBS) were added to each well and incubated for 3 hours at 37 ^o^C in a humidified atmosphere with 5% CO_2_. After this period, 100-mL aliquots of a sodium dodecyl sulfate (SDS) solution in 10% 0.01-N hydrochloric acid (HCl) were added to each well to dissolve the crystals, and the plate was incubated again for 24 hours at 35 ^o^C for further OD readings.^25^ ^,^ ^26^ ^,^ ^27^ The OD was measured in a Thermo Labsystems 352 Multiskan MS microplate reader (Labsystems Oy, Helsinki, Finnland) with a 550-nm filter. Culture medium without cells was the blank. Control wells absorbance (cells cultured in complete medium) was considered as 100% cell viability. Results were expressed as OD.

### Mini-implant surface scanning electron microscopy (SEM) 

In order evaluate qualitatively mini-implants surface characteristics as to the presence of any imperfection and corrosion areas, a sample of each artificial saliva immersion group and a sterile packaged control sample from the same lot were chosen randomly and examined by means of scanning electron microscope (JEOL Model JSM5410, Jeol Ltd, Japan) equipped with energy dispersive spectroscopy (EDS) to analyze surface element composition. Surface topography of the mini-implant head, normally exposed to the oral environment, was examined under 35x and 1000x magnification. 

### Atomic absorption spectrophotometry analysis (AAS) of artificial saliva mini-implant corrosion products 

Mini-implant extract solutions obtained after 30- and 60-day immersion periods in artificial saliva were analyzed with the aid of an atomic absorption spectrophotometer (Aanalyst^TM^ 200, PerkinElmer, Waltham, MA, USA) to determine and quantify the amount of aluminum and vanadium ions released due to corrosion and oxidation. Artificial saliva incubated for 30 and 60 days was used as a control solution (blank). The gas mixture used was air/acetylene. The wavelengths employed were 309.3 nm for aluminum and 313.3 nm for vanadium. The limits of sensitivity for aluminum and vanadium were 1.0 µg/mL and 0.5 µg/mL, respectively.

### Statistical analysis

Results were expressed as a mean ± SEM (standard error of the mean). One-way ANOVA/Bonferroni's post-tests were performed with GraphPad PRISM software (GraphPad Software, Inc., San Diego, CA, USA). Statistical significance was determined at the level of *p* < 0.05.

## RESULTS 

### Cytotoxicity assays

**Figure 2:**
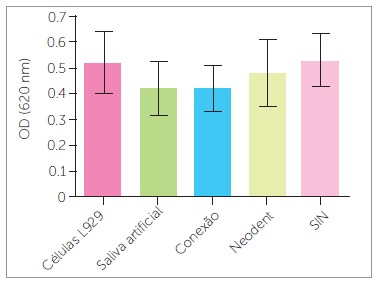
Crystal violet colorimetric assay for L929 cell samples incubated with extract solutions of mini-implants obtained after 30 days of immersion in artificial saliva.

L929 cell morphological analysis under light microscopy showed no cell monolayer destruction. Similarly, the crystal violet assay indicated absence of cell death. A certain optical density decrease was registered for the L929 cell samples incubated with Conexão(tm) and Neodent(tm) mini-implant extract solution, but this decrease was similar to negative control (artificial saliva) and no statistical difference was found among them (*p* = 0.781 and *p* = 0.514 for 30 and 60 days, respectively) (Fig 2).

**Figure 3:**
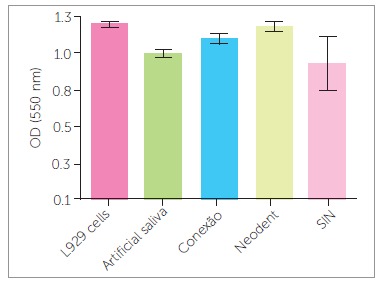
MTT colorimetric assay for L929 cell samples incubated with extract solutions of mini-implants obtained after 60 days of immersion in artificial saliva.

The MTT colorimetric assay demonstrated no cell metabolic activity inhibition for the three mini-implant extract solutions tested, particularly in the 30-day samples. Although SIN(tm) mini-implants led to more cell metabolism alteration than the others in the 60-day period, the difference was not statistically significant (*p* = 0.125 and *p* = 0.273 for 30 and 60 days, respectively) (Fig 3).

### Analysis of mini-implant surfaces by means of SEM

**Figure 4:**
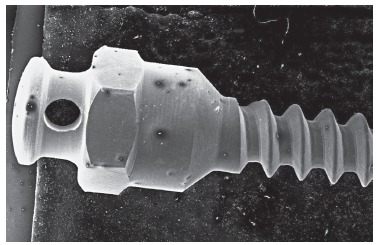
Photomicroscopy of Neodent mini-implant after 60 days of immersion in artificial saliva (50x).

Micro analysis of Neodent(tm) mini-implants demonstrated more adhered particles and a higher number of darkened spots on their surfaces, especially samples immersed for a longer period, as compared to the control group, although these surfaces revealed to be smooth and regular (Fig 4). 

**Figure 5:**
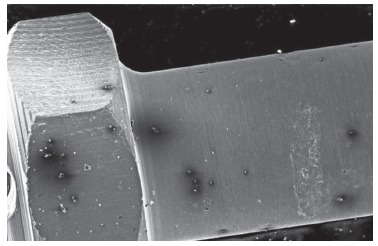
Photomicroscopy of SIN mini-implant after 30 days of immersion in artificial saliva (150x).

SIN(tm) mini-implant analysis of the control group revealed a smooth surface, but with adhered particles in some darkened areas. The artificial saliva immersed sample demonstrated a rough area between the screw body itself and the head, thus suggesting corrosion (Fig 5). 

**Figure 6:**
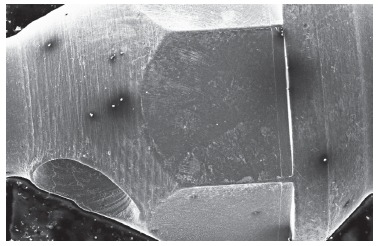
Photomicroscopy of Conexão mini-implant after 60 days of immersion in artificial saliva, evidencing the presence of whitish spots.

Conexão(tm) mini-implant analysis of the control group demonstrated a very smooth surface without significant roughness, without adhered particles or darkened spots. The artificial-saliva-immersed samples remained smooth and free from corrosion, presenting only small amounts of adhered particles and darkened areas, especially after 30 days. The 60-day samples presented some whitish spots, characteristic of calcium buildup (Fig 6). 

The energy-dispersive X-ray spectrometer (EDS) revealed the presence of titanium, aluminum and vanadium, as well as traces of calcium, silicon, potassium, chloride, magnesium and carbon, thus reflecting the artificial saliva composition in all mini-implants tested. 

### Measurement of aluminum and vanadium ions in mini-implant extract solutions by means of atomic absorption spectrophotometry (AAS)

The artificial saliva used solely as control, in both periods, showed no sign of aluminum or vanadium. Similarly, the concentration of Al and V ions in the artificial saliva mini-implant extract solution was below the sensitivity threshold of the equipment, thus demonstrating that whatever amount is released, it is so minimal that it is not detrimental to an individual's health.

## DISCUSSION

In this study, orthodontic mini-implants ready for clinical use as anchorage devices were tested for their potential toxic effect. These devices are manufactured almost exclusively from a titanium alloy (Ti_6_Al_4_V) with the addition of aluminum and vanadium for greater strength and fatigue resistance^12^ ^,^ ^13^ to withstand orthodontic forces for tooth movement. However, aluminum and vanadium have been associated with side effects in the human body.

Results yielded by the present study demonstrated that Conexão(tm), Neodent(tm) and SIN(tm) mini-implant extract solutions obtained after 30- and 60-day immersion periods did not affect cell viability or decreased cell metabolism, thus demonstrating that none of them are cytotoxic. There was no statistical difference among groups (*p* > 0.05). This finding is in agreement with several studies that support the high biocompatibility of titanium and its alloys.^4^ ^,^ ^8^ ^,^ ^10^ ^,^ ^11^ One of the main requirements for a metal or alloy to be biocompatible is the lack of release of corrosion products, which may lead to adverse effects.

According to SEM mini-implant surface analysis, there was no significant corrosion. This result confirms the high corrosion resistance of these mini-implants, even if they are composed of a less resistant alloy compared with other devices, which do not have aluminum and vanadium in their composition. However, all mini-implants immersed for 60 days showed darkened spots and more adhered particles suggestive of decreased corrosion resistance.

Concentrations of aluminum and vanadium ions above 0.2 µg/mL can affect the growth rate of L929 cells. In the present study, AAS analyses, presenting sensitivity thresholds of 1.0 µg/mL and 0.5 µg/mL for aluminum and vanadium, respectively, did not show release of these metals in the extract solutions analyzed. It is worth mentioning that, despite the evidence of good corrosion resistance and biocompatibility of all mini-implants tested, SIN(tm) mini-implant presented rough areas that suggest corrosion or manufacturing defects. The 60-day samples exhibited the greatest alteration in the MTT assay, which is more sensitive than the crystal violet assay.^26^ The combination of these two results calls attention to the corrosion potential of this mini-implant, although the results demonstrated that they were not statistically different.

 The other elements also detected in the alloy were contaminants, such as calcium, potassium, chloride, oxygen, silicon and magnesium; they were probably from artificial saliva or were incorporated during the cleaning and passivation protocols in industrial handling of all mini-implants tested.

Recent studies^28^ ^,^ ^29^ ^,^ ^30^ have demonstrated that although titanium alloys are considered highly corrosion-resistant because of the stable passive titanium oxide layer on their surface, they are not inert to corrosive attack. Retrieved mini-implants showed considerable surface and structural alterations, such as dullness, corrosion, and blunting of threads and tips. Their surfaces showed interactions and adsorption of several elements, such as calcium, at the body region. 

In the present study, taking into consideration that 60 days was the maximum period that the mini-implants were exposed to artificial saliva, a time in which all samples remained static, not submitted to any orthodontic force in which the results demonstrate no signs of corrosion in the mini-implants from all manufacturers, the presence of manufacturing/corrosion defects on the SIN(tm) mini-implants surface causes concern. In studies employing longer immersion periods and friction simulation, these mini-implants most probably would release greater amounts of corrosive products, which could be harmful because the protective oxide layer would be removed from certain areas and, therefore, would not prevent corrosion.^9^ ^,^ ^13^ ^,^ ^29^

Although the corrosion resistance of titanium is well documented in the literature, a gap regarding this matter in mini-implants commonly used in Orthodontics still remains. Therefore, further studies should be performed to clarify corrosion resistance and cytotoxicity of these devices, testing longer periods of immersion, harder wear simulation conditions, pH alterations, and the presence of fluoride ions in the corrosive medium.

## CONCLUSION 

Mini-implants of three commercial brands (Conexão(tm), Neodent(tm) and SIN(tm)) exhibited good corrosion resistance after 30- and 60-day immersion periods in artificial saliva. The release of aluminum and vanadium ions was not detected in the extract solutions analyzed, within the limits of the AAS technique used. No cytotoxicity was observed in L929 cell morphological evaluation, growth inhibition, cell damage, and/or alteration of cellular metabolism.

Orthodontic mini-implants manufactured by Conexão(tm), Neodent(tm) and SIN(tm) present high corrosion resistance and are not cytotoxic.
